# Invasion of sorghum in the Americas by a new sugarcane aphid (*Melanaphis sacchari*) superclone

**DOI:** 10.1371/journal.pone.0196124

**Published:** 2018-04-25

**Authors:** Samuel Nibouche, Laurent Costet, Jocelyn R. Holt, Alana Jacobson, Adrian Pekarcik, Joëlle Sadeyen, J. Scott Armstrong, Gary C. Peterson, Neal McLaren, Raul F. Medina

**Affiliations:** 1 CIRAD, UMR PVBMT, La Réunion, France; 2 Department of Entomology, Texas A&M University, TAMU MS, Texas, United States of America; 3 Department of Entomology and Plant Pathology, Auburn University, Auburn, Alabama, United States of America; 4 Wheat Peanut and Other Field Crops Research, USDA-ARS, Stillwater, Oklahoma, United States of America; 5 Texas A&M AgriLife Research and Extension Center, Lubbock, Texas, United States of America; 6 Natural and Agricultural Sciences, University of the Free State, Bloemfontein, South Africa; Sichuan University, CHINA

## Abstract

In the United States (US), the sugarcane aphid (SCA) *Melanaphis sacchari* (Zehnter) (Hemiptera: Aphididae) was introduced in the 1970s, however at that time it was only considered a pest of sugarcane. In 2013, a massive outbreak of *M*. *sacchari* occured on sorghum, resulting in significant economic damage to sorghum grown in North America including the US, Mexico, and Puerto Rico. The aim of the present study was to determine if the SCA pest emergence in American sorghum resulted from the introduction of new genotypes. To this end we used microsatellite markers and COI sequencing to compare the genetic diversity of SCA populations collected in the Americas after the 2013 SCA outbreak on sorghum (during 2013–2017) to older samples collected before the pest outbreak (during 2007–2009). Our results show that the SCA outbreak in the Americas and the Caribbean observed since 2013 belong to populations exhibiting low genetic diversity and consisting of a dominant clonal lineage, MLL-F, which colonizes *Sorghum* spp. and sugarcane. The comparison of MLL-F specimens collected post-2013 with specimens collected in Louisiana in 2007 revealed that both populations are genetically distinct, according to COI sequencing and microsatellite data analyses. Our result suggest that MLL-F is a new invasive genotype introduced into the Americas that has spread rapidly across sorghum growing regions in the US, Mexico, Honduras and the Caribbean. The origin of this introduction is either Africa or Asia, with Asia being the most probable source.

## Introduction

The sugarcane aphid (SCA) *Melanaphis sacchari* (Zehnter) (Hemiptera: Aphididae) (S1 Fig) is a major pest of sorghum in many areas of Africa, Asia, Australia, the Far East, and parts of Central and South America [1, 2]. In the United States (US), *M*. *sacchari* was introduced in the 1970s [3,4], however at this time it was considered a pest of sugarcane only, causing damage to this crop mostly as a virus vector [5]. In 2013, massive outbreaks of *M*. *sacchari* on sorghum resulting in economic damage began in North America [6]. Today, twenty US states and all sorghum producing regions of Mexico have experienced heavy SCA infestations on sorghum since the 2013 pest outbreak [7,8], while populations on sugarcane remain moderate [9]. This sudden change of the SCA pest status in the US and Mexico, from a minor pest of sugarcane to a major pest of sorghum, has led to the hypothesis of either (i) an introduction in North America of a sorghum specialized SCA biotype or (ii) of a shift in host preference in pre-existing local SCA populations. Similar to numerous other aphid studies, which report aphids exhibiting host plant specialization among their populations [10–13], a recent study has demonstrated the existence of sympatric sorghum and sugarcane biotypes in *M*. *sacchari* on Reunion Island (Indian Ocean) [14]. The occurrence of sympatric biotypes in other areas has been assessed, but not been found [9].

In a population genetics study carried out using microsatellite markers [15] and COI barcoding, Nibouche et al. [16] showed that *M*. *sacchari* populations collected at a worldwide scale during 2002–2009 were organized in five multilocus, or clonal, lineages (MLL) structured according to geography. Four lineages were observed outside the US: MLL-A was observed in Africa, MLL-B in Australia, MLL-C in South America, Caribbean, East Africa and Indian Ocean islands, MLL-E in China. The US samples reported by Nibouche et al. [16] were collected primarily from sugarcane in 2007 in Louisiana and Hawaii and all belonged to the MLL-D lineage, which was observed nowhere outside the US. Considering that *M*. *sacchari* was reported in Hawaii since the 19^th^ century [17], this suggested a Hawaiian origin of the populations introduced into the continental US in the 1970s [16].

Since the SCA outbreak on US sorghum, two recent studies have dealt with the genetics of post-2013 SCA populations in the US. In a first study, Medina et al. [9] used AFLP markers to examine the genetic similarity of SCA specimens collected from eight US states on sugarcane, sorghum and Johnsongrass and found that SCA were grouped within three genetically distinct clusters, although there was no evidence of host plant or geographic population structure. In a second study, Harris-Shultz et al. [8] developed 38 new SCA specific microsatellite markers. Adding these 38 markers to 14 previously published ones [15], they studied the genetic diversity of specimens collected on sorghum from 17 locations of Southern US states and Puerto Rico. Their results revealed a very low genetic diversity and showed that almost all samples belong to one ‘superclone’ genotype [18]. Similarly, they examined the genetic diversity of the obligate aphid symbiont *Buchnera aphidicola* using 12 microsatellite markers and the same pooled aphid samples from the genotyping experiment markers showed a lack of genetic diversity among *Buchnera*. Both studies used different strategies for their genetic analysis, genotyping of individual specimens by Medina et al. [9] versus pooling of DNA from several specimens by Harris-Shultz et al. [8], which could explain why they did not revealed a similar level of genetic differentiation.

Studies by Medina et al. [9] and Harris-Shultz et al. [8] provide insight into the genetic diversity of the current sorghum infesting SCA populations and did not reveal any host-associated differentiation or geographic genetic structure. However, because they did not use the same molecular markers as the ones used to characterize pre-2013 populations [16] neither study could determine if post-2013 US SCA populations were genetically distinct from the populations previously described by Nibouche et al. [16]. The aim of the present study was to determine whether SCA pest emergence is due to the possible introduction of new genotypes. To do this we compared the genetic diversity of SCA populations collected in the Americas after the 2013 SCA outbreak on sorghum (during 2013–2017) to older samples collected before the pest outbreak (during 2007–2009) using the same COI and microsatellite markers used previously [16].

## Materials and methods

### Sampling

A ‘specimen’ refers to one individual aphid and a ‘sample’ refers to several specimens collected from the same host plant species at a given location on the same date. Only a few aphids were collected on each plant sampled to avoid collecting several individuals from the same colony. Aphids were placed in 95% ethanol in the field, and stored at -80°C. Sampling aphids in these locations did not require specific permissions and these aphids were not endangered or protected species.

This study comprised a total of 544 specimens (189 specimens collected post-2013 across the continental US and 355 specimens collected in Louisiana in 2007 among which 243 were analyzed in our previous study [16]). The 189 specimens from 106 samples collected post-2013 includes individuals collected from Louisiana (n = 37), Texas (n = 24), Oklahoma (n = 19), Arkansas (n = 4), Georgia (n = 9), Alabama (n = 23), Tennessee (n = 14), Florida (n = 6), California (n = 4), Mexico (n = 5), Haiti (n = 12), Guadeloupe (n = 8), Puerto Rico (n = 20), and Peru (n = 4). Specimens collected in 2007 from Louisiana included 30 samples, 29 from sugarcane and one from Johnsongrass; eight of them were previously analyzed in [16]. Specimens collected post-2013 across the Americas included 34 samples from *Sorghum bicolor*, 21 samples from Johnsongrass, one sample from maize and 20 samples from sugarcane (S2 Fig). A complete sample list including collection information for each sample is provided in S1 Table. The list of the specimens included in the present study is provided in S2 Table.

### DNA extraction

DNA of specimens analyzed in our previous study [16] were extracted using the ‘‘salting-out” protocol of Sunnucks and Hales [19]. The DNA extraction of the 301 new specimens analyzed in this study were conducted either with a destructive or a non-destructive DNA extraction using the Qiagen DNeasy Blood & Tissue Kit (Qiagen, Courtaboeuf, France). Destructive DNA extraction was performed according to the manufacturer’s protocol with modifications; the aphid was ground in a 1.5 mL Eppendorf tube with a TissueLyser II, Qiagen using glass beads for 3 min at 30 Hz, in 20 μL of the ready-to-use proteinase K solution of the Qiagen DNeasy Blood & Tissue Kits. The remainder of the extraction was done according to the manufacturer's instructions. The non-destructive DNA extraction allowed specimens to be analyzed for both microsatellite and morphometrics by slide mounting (‘lame’ mentioned in S2 Table, column field code). Non-destructive DNA extraction was performed according to the manufacturer’s protocol and the insect body was retrieved from the first elution column for slide mounting (potassium chloride treatment, followed by a chloral hydrate plus phenol treatment).

### COI

We used LCO 1490 / HCO 2198 Folmer et al. primers [20] to generate COI sequences for all SCA specimens. PCR was carried out using the protocol of Kim and Lee [21]. PCR products were purified and bidirectionally sequenced using Sanger sequencing. Sequence alignments were performed using Geneious software version 10.0.5 [22]. Most sequences were 658 bp long; sequences less than 500 bp long were not included in analysis. According to Genbank, to published data and to the present study, all known substitutions in the *M*. *sacchari* COI sequence are located between 95 bp and 531 bp (Table 1). Therefore, specimens with incomplete sequences between the 95 bp and the 531 bp COI sequence were discarded from the analysis.

**Table 1:** *M*. *sacchari* COI haplotypes.

COI haplotype	position (bp)					
	95	263	294	343	392	531
H1	A	C	G	A	A	C
H2	A	T	G	G	A	C
H3	A	C	A	G	A	C
H4	G	C	A	G	A	C
H5	A	C	G	A	G	C
H6	A	C	G	A	A	A

Position and nature of nucleotide substitutions in *M*. *sacchari* COI sequence from this study (haplotype H6), from published data (H1, H2, H3) [14, 28, 29], and from Genbank (H4 = JX051388, JX051389, JX051390; H5 = HQ112185, JX051402).

The analysis included 105 COI sequences generated in the present study, and 9 from a previous study [16], which corresponded to 68 samples (S2 Table). Sequences were deposited in BOLD and Genbank for reference (S2 Table). COI haplotypes were identified by comparing sequence similarity; identical sequences were assigned the same haplotype. A haplotype network to identify sequence similarity among all collected haplotypes was computed using PHYLOViZ 2.0 software [23].

### Microsatellites

Nine microsatellite markers were selected among the 14 developed by our team [15]. PCR reactions were performed with labelled primers and multiplexed as described by Nibouche et al. [16]. Genotyping was carried out using an ABI PRISM 3110 and alleles were identified at each locus by comparison with a size standard using Gene-Mapper version 2.5 software (Applied Biosystems). The total microsatellite genotyping dataset included 526 specimens (S2 Table): 239 specimens from 2007 Louisiana collections that were analyzed previously [14], and 287 new specimens genotyped for this study (110 additional specimens collected from Louisiana in 2007, and 177 specimens collected across the Americas during and after 2013, including 35 specimens collected in Louisiana). Single combinations of alleles were characterized and arranged as distinct multilocus genotypes (MLG) (Table 1).

Population genetic structure of all the individuals screened using microsatellites was examined using two different methods. The first method used the number of allele differences among specimens as a metric to compute a distance matrix among MLGs using the R package RClone 1.0.2 [24]. This genetic distance matrix was used to compute a minimum spanning network with HAPSTAR [25]. If the MLGs were separated by less than two stepwise mutations, they were grouped together into a single multilocus lineage (MLL). The second method consisted of a factorial discriminant analysis in GENETIX [26], which is a multivariate analysis approach that uses no a priori genetic assumptions for relationships between allelic differences and genetic distance.

To detect the signature of asexual reproduction, we used RClone [24] to compute p_sex_, the probability that the occurrences of repeated MLGs originated from distinct sexual reproductive events. A p_sex_ value lower than 0.01 supports the hypothesis that two specimens with the same multilocus genotype are part of the same clone, and, therefore, unlikely to be derived from distinct sexual reproductive events [27].

### Maps

The maps synthesizing some of the results were drawn using QGIS 2.18 (www.qgis.org). The maps of the administrative boundaries of the countries and states were uploaded from the database of Global Administrative Areas GADM 2.8 (www.gadm.org). To locate the samples on the maps, we used the geographic coordinates provided by the people who collected the samples (see acknowledgment section). When the coordinates were missing, we used Google Earth to retrieve the coordinates of the closest location mentioned by the collector. The geographic localisation of the samples is available in the Arthemis database (www.arthemisdb.supagro.inra.fr).

## Results

When COI barcodes were used to characterize SCAs collected from the continental US in sorghum, Mexico and the Caribbean post-2013, most of the specimens were determined as haplotype H1, whereas in 2007 the sole haplotype observed in Louisiana was H3 [16]. Moreover, microsatellite analysis revealed that most of SCA populations collected post-2013 belonged to a newly described MLL (i.e. MLL-F). The MLL observed in Louisiana samples in 2007 (i.e. MLL-D) was only observed in a few post-2013 samples.

### COI analysis

**Fig 1:**
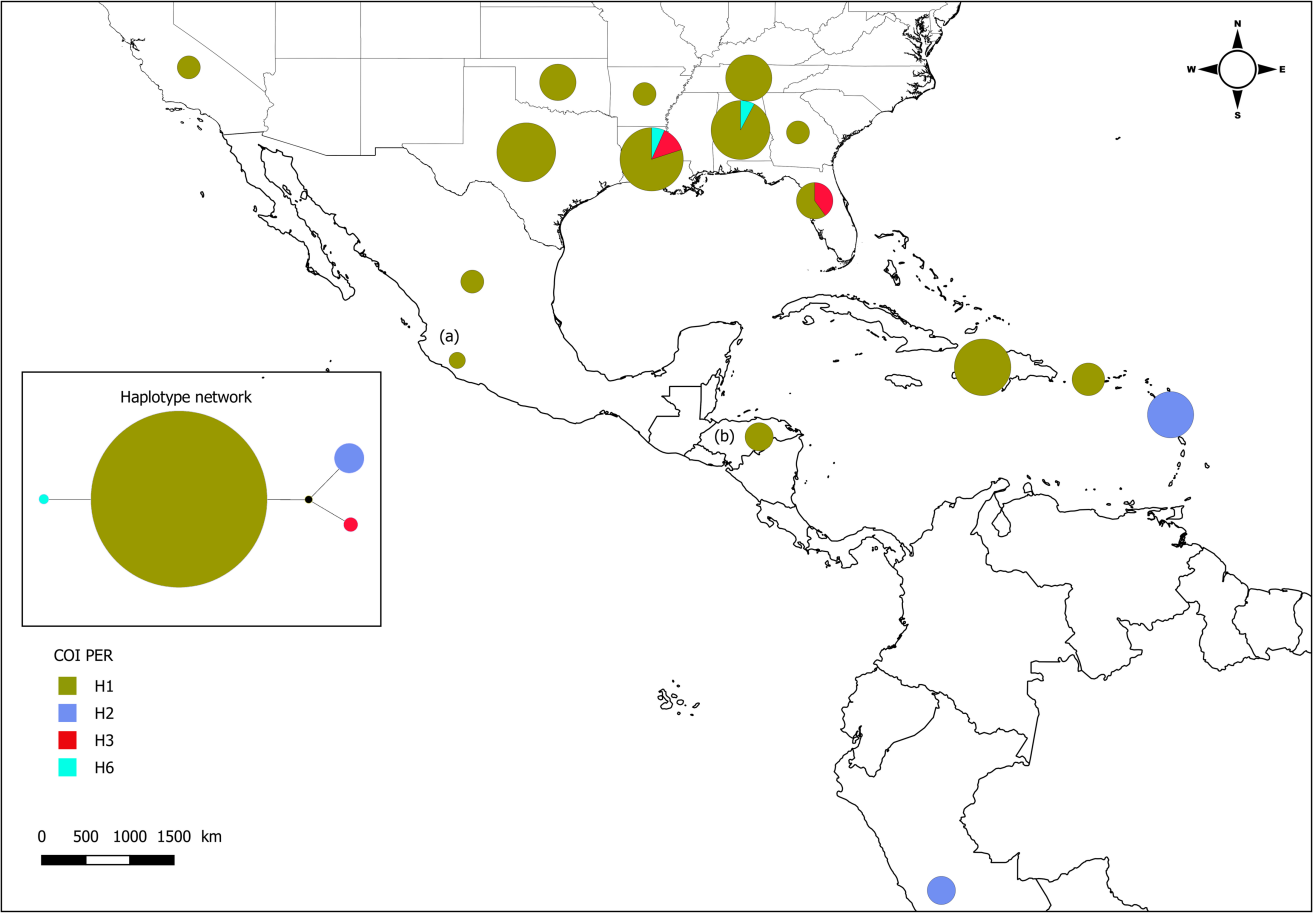
Map of the distribution and relative abundance of the COI haplotypes in post-2013 SCA populations. Aggregation by state, the surface of the pie charts is proportional to the number of genotyped specimens. Haplotype network computed with Phyloviz (embedded figure left): the surface of the circles is proportional to the relative abundance of the haplotype, each node or step represents one base substitution, and the black dot corresponds to an unobserved haplotype. (a) data from Berlanga-Padilla et al. [28]; (b) J. Orozco personal communication.

The analysis of the 114 COI sequences yielded four haplotypes: H1, H2, H3 and H6 (Table 1, Fig 1). All 2007 Louisiana collections belong to haplotype H3 (31 specimens). The majority of specimens collected from US and Mexico post-2013 belonged to haplotype H1 (61 / 67 = 91% of specimens), which has not previously been observed in the Americas before 2013.

Indeed, haplotype H1 has only been identified before 2013 in samples collected from Africa, China, and Australia [16, 29]. Two other haplotypes were also observed post-2013: (i) H3 in Louisiana and Florida (three specimens from sugarcane, one specimen from sorghum), which was the sole haplotype observed in the US in 2007; and (ii) haplotype H6 in Louisiana and Alabama (one specimen from Johnsongrass and one from sugarcane), which was not observed before. Haplotype H1 was also observed in Puerto Rico, Haiti, Honduras, and Southern Mexico, while haplotype H2 was observed in Guadeloupe and Peru. Previously H2 was observed in South America and French Lesser Antilles [16]. According to data provided by J. Orozco (personal communication), haplotype H1 was also observed from three specimens collected in 2016 in Honduras (Fig 1). Data from Berlanga-Padilla et al. [28] also confirm the presence of H1 in a specimen collected in Mexico in 2016 (Fig 1).

### Microsatellite analysis

The genotyping of the post-2013 specimens with nine microsatellite loci identified 12 multilocus genotypes (MLGs) (Table 2). Among these 12 MLGs, only two of them (Ms11 and Ms9) were already observed during the Nibouche et al. study [16]. One MLG, Ms10, was observed in low frequency in 2007 [16] but not recovered post-2013. Calculation of p_sex_ for the five MLGs that were encountered in at least two samples (i.e. Ms9, Ms11, Ms24, Ms50, Ms53, Ms57) yielded values ranging from 0.0003 to < 0.0001, confirming that all replicates from the same MLG were unlikely the result from distinct zygotes, but were the result of clonal reproduction [27].

**Table 2:** List of MLGs observed in this study.

MLL	MLG	Microsatellite locus								
		CIR-Ms-G08	CIR-Ms-G403	CIR-Ms-C08	CIR-Ms-G01	CIR-Ms-E01	CIR-Ms-G12	CIR-Ms-E03	CIR-Ms-D02	CIR-Ms-G02
MLL-C	Ms11	233/233	251/259	197/199	185/210	247/247	212/216	186/193	228/232	199/199
	Ms24	233/233	251/259	197/199	185/210	247/247	212/218	186/193	228/232	199/199
MLL-D	Ms9	233/233	251/259	197/199	185/206	247/247	212/216	186/188	226/234	201/201
	Ms102	233/233	251/259	197/201	185/206	247/247	212/216	186/188	226/234	201/201
	Ms10	233/233	251/259	197/199	185/206	247/247	212/218	186/188	226/234	201/201
MLL-F	Ms50	227/229	253/253	199/203	198/198	245/245	212/216	174/174	220/222	199/199
	Ms51	227/227	253/253	199/203	198/198	245/245	212/216	174/174	220/222	199/199
	Ms52	227/229	251/253	199/203	198/198	245/245	212/216	174/174	220/222	199/199
	Ms53	227/227	255/255	199/203	198/198	245/245	212/216	174/174	220/222	199/199
	Ms54	227/229	255/255	199/203	198/198	245/245	212/216	174/174	220/222	199/199
	Ms55	227/229	253/253	199/203	198/198	245/245	216/216	174/174	220/222	199/199
	Ms56	227/227	255/255	203/203	198/198	245/245	212/216	174/174	220/222	199/199
	Ms57	227/227	253/253	197/201	198/198	245/245	212/216	174/174	220/222	199/199

Allele sizes (bp) at nine microsatellite loci.

**Fig 2:**
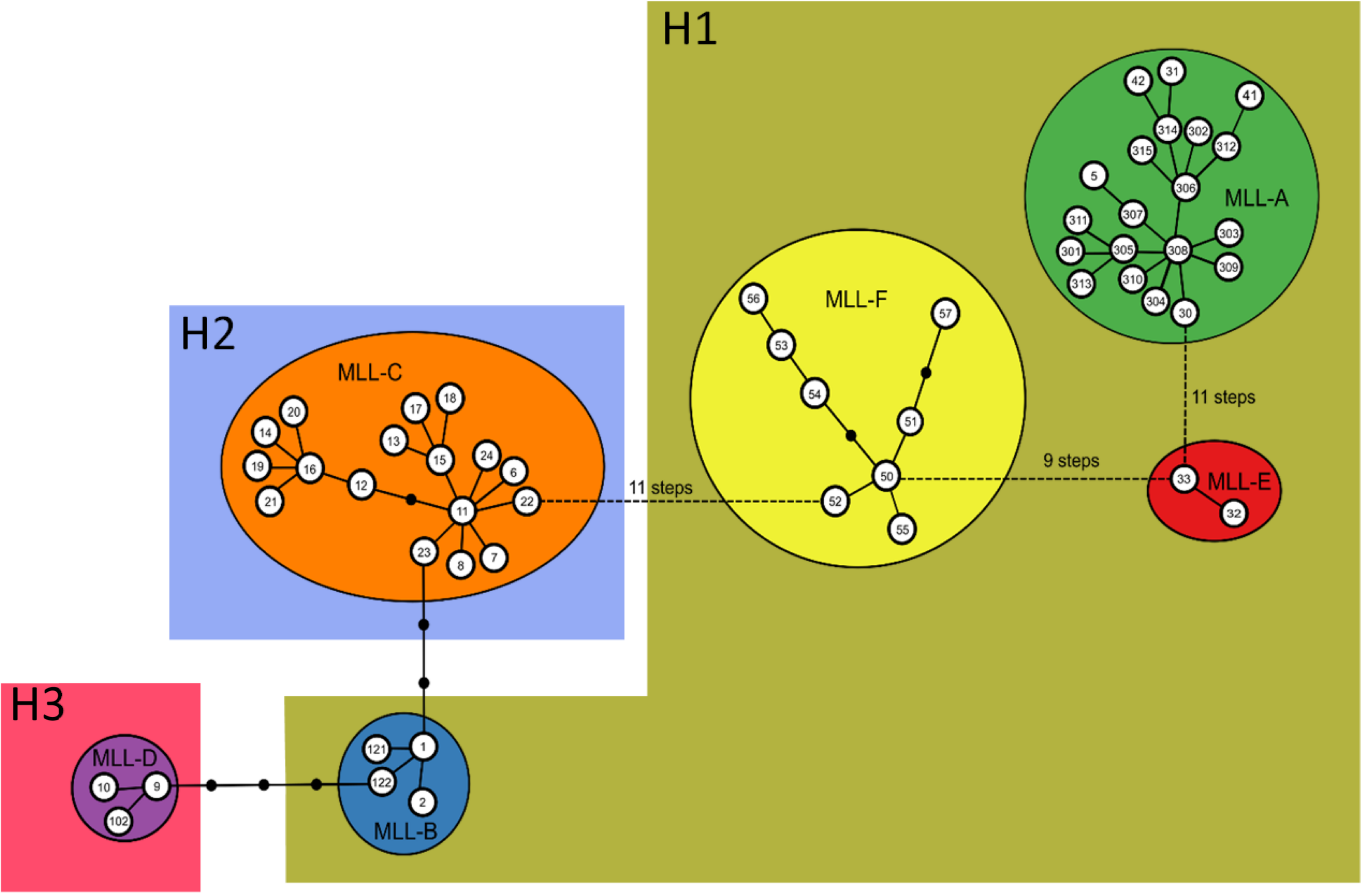
Minimum spanning network computed with Hapstar based on the genotyping at nine microsatellite loci. MLGs are either from the present study or from previous studies [14,16]. Numbers within the circles refer to the MLG number according to [14,16] and the present study. MLGs in the same colored box share the same COI haplotype: H1, H2 or H3. Haplotype H6, which was observed in two Ms50 specimens, is not represented. The steps between each MLL represent the number of allele differences.

The minimum spanning analysis identified six multilocus lineages (MLLs) including five previously described MLLs [16] and a new MLL-F (Fig 2). MLL-F appears widely separated (9 or more allele differences) from the five MLLs previously described at the worldwide scale. The new MLL-F is comprised of eight MLGs (Ms50 to Ms57) collected post-2013 only, in the US, Mexico, Haiti and Puerto Rico from sorghum, Johnsongrass, maize, and sugarcane. The five MLGs belonging to MLL-F were separated by up to five allele differences. Within MLL-F, MLGs form a subnetwork with less than two allele differences between each node.

Among the MLGs belonging to MLL-F, Ms50 was predominant, representing 90% (147 /163) of the MLL-F specimens. By decreasing order of relative frequency were Ms57 (4% of the MLL-F specimens), Ms53 (3% of the MLL-F specimens), Ms51, Ms52, Ms54, Ms55 and Ms56 (one specimen each). In addition, two new MLGs were identified, Ms24 (Peru, sugarcane) and Ms102 (Louisiana, sugarcane), that grouped with MLL-C and MLL-D respectively.

**Fig 3:**
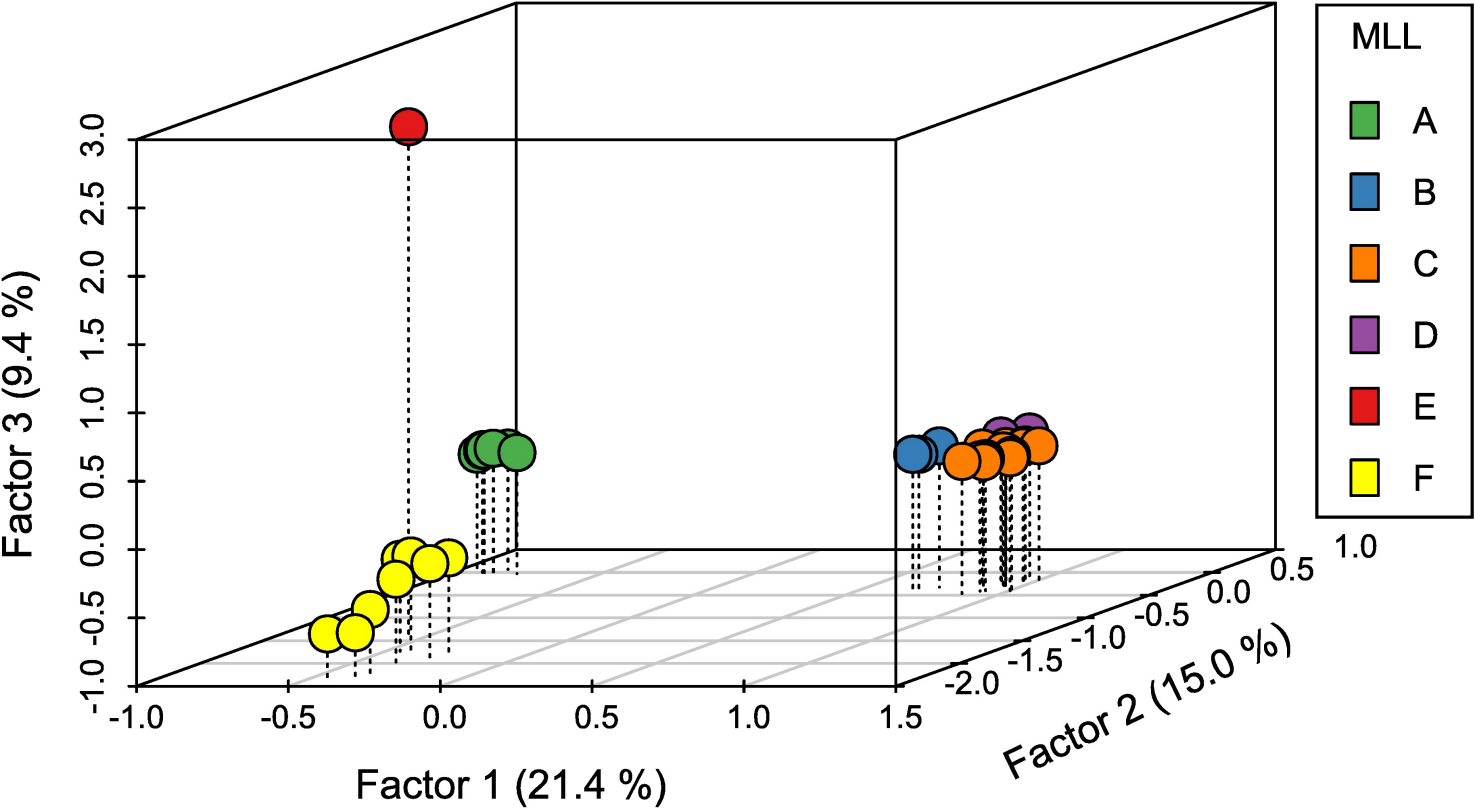
Factorial components analysis of the genetic diversity of the 54 MLGs using genotypic data at nine microsatellite loci. The graph was produced with R scatterplot3D package [30].

The analysis of the genetic diversity of the 54 MLGs using a factorial component analysis with GENETIX provides support for the results of the minimum spanning network (Fig 3). In this analysis, genetic variation partitioned across the three axes shows that individuals from all identified MLLs are genetically differentiated from each other, and confirms that MLL-F is genetically distinct from the five MLLs previously described at the worldwide scale. The genetic similarity observed between MLLs in this factorial component analysis also generally reflects the number of allele differences calculated in the minimum spanning network; MLLs with fewer allele differences in the minimum spanning network are more genetically similar to each other than MLLs separated by a larger number of allele differences.

**Fig 4:**
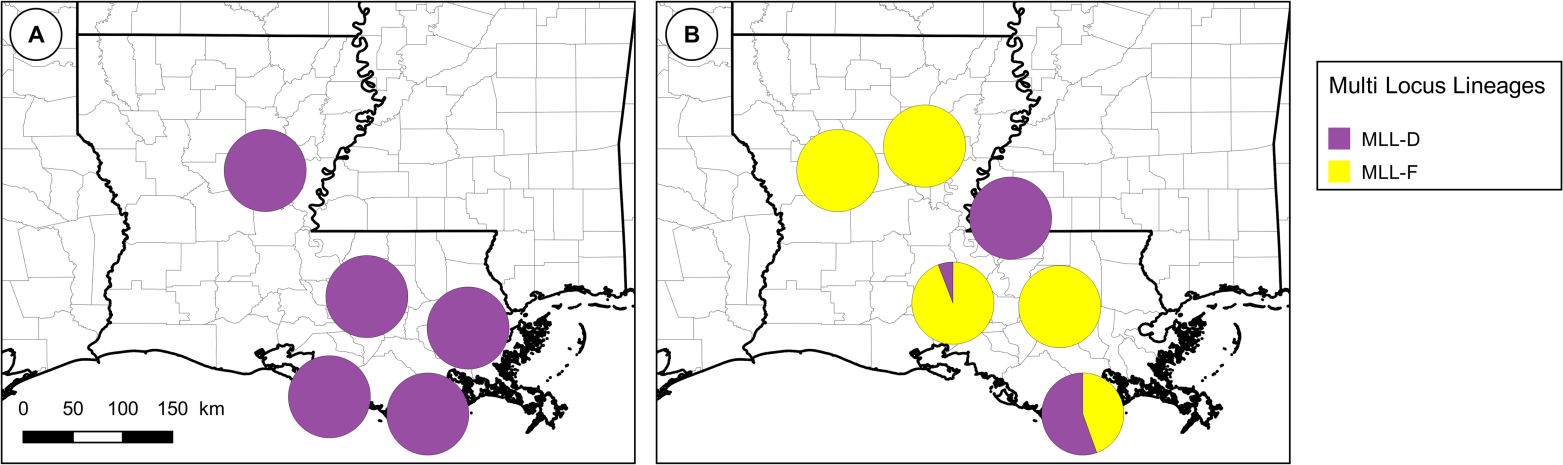
**Evolution of the distribution of MLL in Louisiana from 2007 (A) to 2013–2017 (B).** Data are summarized by parish. The size of the pie-charts is not proportional to the number of genotyped specimens.

In Louisiana (Fig 4), which was the only continental US state studied both in our previous and present study, a marked change occurred between 2007 (Fig 4A) and 2013–2016 (Fig 4B). In 2007, all genotyped specimens (349 specimens from 30 samples) belonged to MLL-D. In 2013–2016 most of the specimens belonged to MLL-F (32 specimens from 13 samples), and three of them (from two samples) belonged to MLL-D. These three MLL-D specimens from 2013–2016 were all collected on sugarcane.

**Fig 5:**
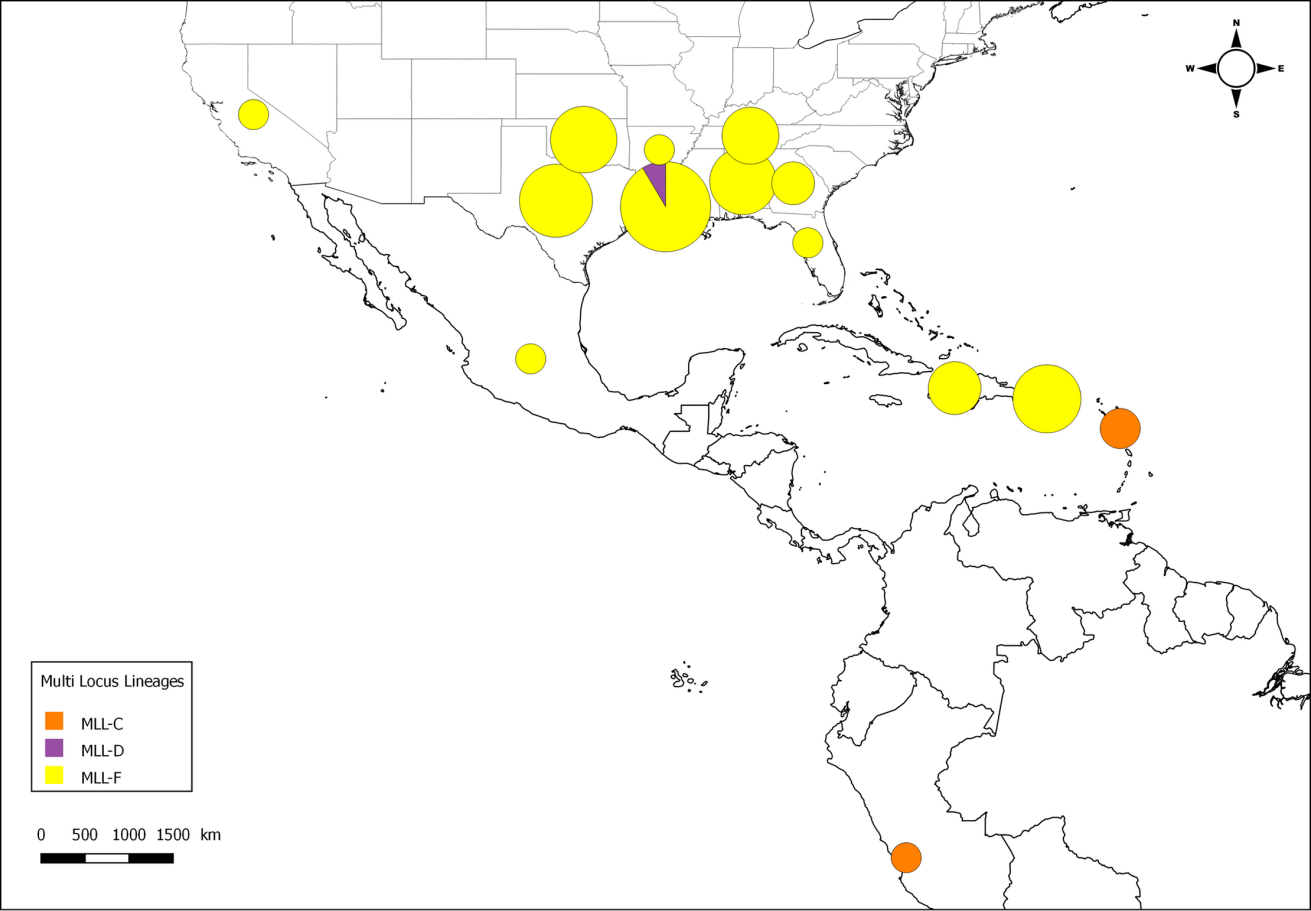
Distribution map of the microsatellite multi locus lineages (MLLs) in post-2013 samples. The size of the pie charts is proportional to the number of specimens.

At the continental scale, the distribution of the MLLs in 2013–2017 is shown in Fig 5. MLL-F appears dominant everywhere in the sorghum growing regions of the continental US; it is also present in Mexico, Puerto Rico and Haiti. MLL-D is only observed in Louisiana. In Guadeloupe and Peru, MLL-C is observed, as it was previously observed in South America and the Caribbean in 2007–2009 [16].

**Fig 6:**
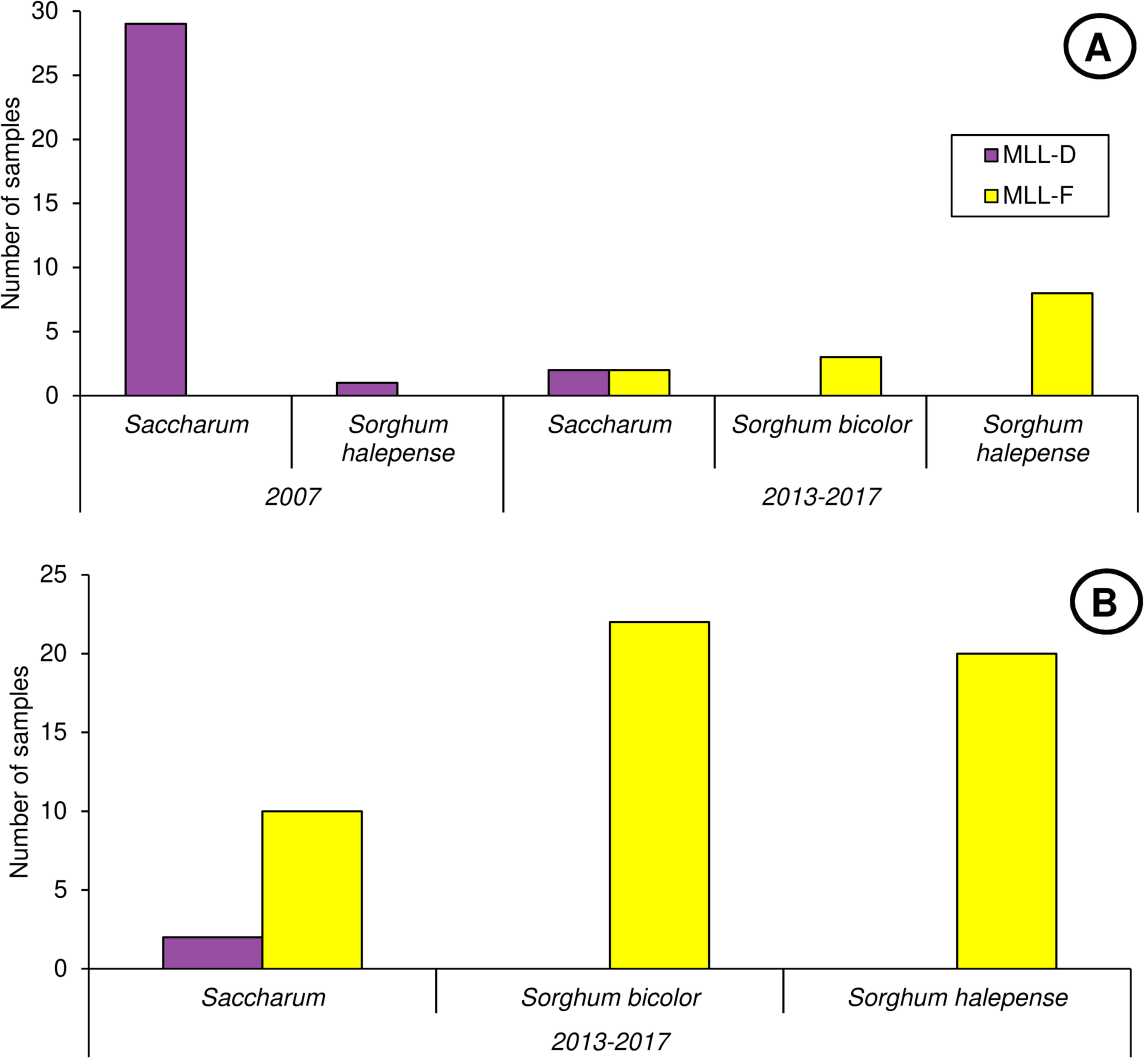
**Prevalence of MLL-C and MLL-D on different host plant species in Louisiana (A) and in the continental US (B).** A: Prevalence (number of samples bearing at least one specimen of each of the MLLs described in this study) of MLL-D and MLL-F in Louisiana among 2007 or post-2013 samples, according to host plant. B: prevalence of MLL-D and MLL-F in the continental US among post-2013 samples.

The prevalence of MLL-C and MLL-D on different host plant species is summarized in Fig 6. Globally, MLL-D was mostly observed on sugarcane, except for one Johnsongrass sample in 2007, whereas MLL-F exhibited no preference and was observed both on *Sorghum* spp. (grain sorghum, Johnsongrass, sweet sorghum and hay grass) and on sugarcane. Despite its ability to colonize sugarcane, MLL-F was not observed in the 29 Louisiana 2007 samples which were collected from sugarcane.

## Discussion

Our study shows that the SCA outbreak in the Americas and the Caribbean observed since 2013 belong to populations exhibiting low genetic diversity and consisting of a dominant clonal lineage, MLL-F, which colonizes *Sorghum* spp. and sugarcane. The comparison of MLL-F specimens collected post-2013 with specimens collected in Louisiana in 2007 revealed that both populations are genetically distinct, according to COI sequencing and microsatellite data analyses.

Our analysis of microsatellite data is consistent with the results obtained by Harris-Shultz et al. [8]: the post-2013 populations exhibit a low genetic diversity and consist of a dominant MLG (i.e., Ms50) in most of our samples from the continental US, Mexico, Puerto Rico and Haiti. In a recent analysis of the genetic diversity of SCA populations from the continental US using AFLP markers, Medina et al. [9] showed that aphid specimens were grouped within three genetically distinct clusters, without evidence of host plant or geographic population structure. In our study, genetic differentiation revealed by microsatellite markers is observed among MLLs but not within; our 9 microsatellite markers did not detect genetic differentiation within MLL-F as characterized by Medina et al. [9]. We hypothesize that AFLP markers allow a finer analysis of the genetic diversity and that the three AFLP groups could be sub-groups within MLL-F. Alternatively, one may hypothesize that the AFLP groups detected by Medina et al. [9] could correspond to the genetic differentiation of the three COI haplotypes identified in the US. However, when DNA from the Medina et al. [9] study was genotyped with our microsatellites no match between AFLP clusters and microsatellite MLGs was found. Since the congruence between COI haplotypes and microsatellite MLGs is strong, the lack of matching between AFLP clusters and microsatellite MLGs makes the correspondence between AFLP clusters and COI haplotypes unsubstantiated. MLL-F appears as a ‘superclone’ [18], a concept used in aphids when a few asexual genotypes of the same species are able to colonize a wide geographical or ecological range of habitats [31,32]. While sexual morphs have been observed in Mexico [33], our study did not support the occurrence of sexual reproduction events in the geographic range studied, according to the low p_sex_ value observed, which indicates that replicates from the same MLG were unlikely to have derived from distinct reproductive events [27].

In Louisiana, the comparison of the samples collected in 2007 with the samples collected in 2013–2017 revealed a major shift in the genetic composition of SCA populations. First, the COI haplotype H3 observed in 2007 was almost completely replaced by the H1 haplotype. H3, which was the unique haplotype observed in 2007 in Louisiana (among 20 genotyped specimens), was only observed in two specimens among 15 genotyped specimens collected on sugarcane in Louisiana in 2013–2016. Second, MLL-F, which was absent in the 2007 Louisiana samples despite extensive sampling (30 samples, 355 specimens) was very common in the Louisiana post-2013 samples (16 samples, 37 specimens). As MLL-F appeared able to colonize both *Sorghum* spp. and sugarcane, one can conclude that this change was not the result of sampling bias related to a change in the relative abundance of sugarcane versus sorghum host plants between 2007 and 2013–2016 samples, but instead the consequence of the introduction of a new clonal lineage in this region.

If the change in MLLs observed in Louisiana is representative of what occurred across the US, Mexico and the Caribbean, it suggests that MLL-F is a new invasive lineage introduced into the Americas that has spread rapidly across the US sorghum growing regions, in Central America (Mexico and Honduras) and in the Caribbean. The expansion of this MLL is probably still under progress. We did not detect MLL-F in Guadeloupe (among sugarcane samples) or in Peru (in one sugarcane sample), however, an increase of SCA outbreaks has been observed in 2016 in Nicaragua (G. Trouche, pers. comm.), which may indicate that MLL-F has reached this country. This suggests that in 2016 the invasion front was located in Central America somewhere between Nicaragua and Peru, and in the Caribbean somewhere between Puerto Rico and Guadeloupe. The lack of dense sampling in these areas prevented us from precisely locating the front. The range expansion potential of MLL-F in Central and South America and the Caribbean is currently not predictable, but it is probable that the expansion of MLL-F may continue south and begin causing damage in sorghum production regions in Central and South America.

The geographic origin of MLL-F is not evident from our study. From COI data, MLL-F specimens belong to haplotype H1, whose distribution area was previously reported in Africa, Asia and Australia. It is interesting to underline that in Africa and India, which are included in the previous area of distribution of H1, *M*. *sacchari* has been a major pest of sorghum for decades [1]. The introduction of H1 in the Americas coincides with the pest status change of *M*. *sacchari*, from a minor pest of sugarcane to a major pest of sorghum. From microsatellite data, MLL-F appears genetically distant from MLL-B whose distribution area is Australia, from MLL-C whose distribution area is South America, the Caribbean, Indian Ocean and East Africa, and from MLL-D, which was previously observed in Hawaii and Louisiana [16]. MLL-F appears also genetically distant from MLL-A (Africa) and MLL-E (China), but samples of MLL-F have not been collected before this study. The genetic diversity of SCA populations in Africa appears relatively homogeneous and it is unlikely that MLL-F originated there. Indeed, with the exception of few MLL-C specimens detected in Kenya, in our previous work [16], MLL-A was the sole lineage observed in West (Niger, Benin), Central (Cameroon) and East Africa (Kenya). Additional samples from Uganda and South Africa were also genotyped during the present study and belonged to MLL-A as well (data not shown), suggesting that MLL-A is present across the whole African continent. On the other hand, the genetic diversity of SCA populations from Asia has not been widely studied to date, although the *Melanaphis* genus is considered of Asiatic origin [34]. Consequently, it is more probable that MLL-F has an Asiatic origin than an African origin, but further studies are needed to confirm this hypothesis, particularly to investigate the genetic diversity of *M*. *sacchari* in Asia. Such intercontinental introductions of exotic aphid species are not rare. For example in Hawaii there are numerous introductions of aphid species such as the cotton aphid *Aphis gossypii* (Glover) and the taro root aphid *Patchiella reaumuri* (Kaltenbach) [35], while in Florida and the southeastern US eleven newly established aphid species were reported in the 1998–2000 period [36]. In Chile, the introduction of pea aphid *Acyrtosiphon pisum* Harris populations were found to be associated with different host plants as a result of independent introductions of genetically distinct lineages [37]. Another example is the introduction of the Nearctic yellow sugarcane aphid *Sipha flava* (Forbes) in the Palearctic region and its subsequent expansion in the continental Afrotropical region [38–40]. In Florida (US) the introduction of a previously unknown aphid related to SCA was described in 1996 by Halbert and Remaudière [41] as *Melanaphis sorini* Halbert & Remaudière, which originated from East Asia and was introduced through the importation of ornamental grasses (*Miscanthus* sp.). It is therefore possible that the shift in preponderance from MLL-D in 2007 to MLL-F post-2013 is the result of a recent introduction from another country, rather than inadequate sampling of pre-2013 specimens.

As in our previous study [16], the use of COI barcodes did not add new information to resolve the taxonomic ambiguity in the *sacchari* group. Currently, the *sacchari* group is considered by several authors as a regrouping of two species, *M*. *sacchari* which would be preferentially associated with sugarcane and *M*. *sorghi*, which prefers sorghum [42, 43]. Although there is no marked separation by COI barcode sequences in *Melanaphis* specimens analysed by [16], a hypothesis was raised by R.L. Blackman [43] that the two groups formed on one hand by MLL-B, MLL-C and MLL-D and on the other hand by MLL-A and MLL-E could be respectively *M*. *sacchari* and *M*. *sorghi*. This consequently suggests that MLL-F could be *M*. *sorghi*. A complicating factor in clarifying taxonomic classifications is that MLLs can be collected on multiple hosts, and there is limited information about host plant preference and fidelity among these genetic groups. Ongoing additional molecular, morphological, biological and ecological studies are needed to clarify classification of these species. Future studies also need to include genetic characterizations of the populations under study to begin to document genotypic and phenotypic differences among these groups.

## Supporting information

S1 Fig *Melanaphis sacchari* habitus. Apterous female left, neonate larva right (photography by A. Franck).
(TIF)

S2 Fig Geographic distribution of the 106 samples included in this study. (TIF)

S1 Table List of samples. (XLSX)

S2 Table List of specimens with their microsatellite genotype and Genbank and BOLD COI accessions. (XLSX)
