# Supplementary figures and images for: Invasion of sorghum in the Americas by a new sugarcane aphid (Melanaphis sacchari) superclone
Source: PLoS One. 2018 Apr 25;13(4):e0196124. doi: 10.1371/journal.pone.0196124 (PMC5919042; doi:10.1371/journal.pone.0196124)

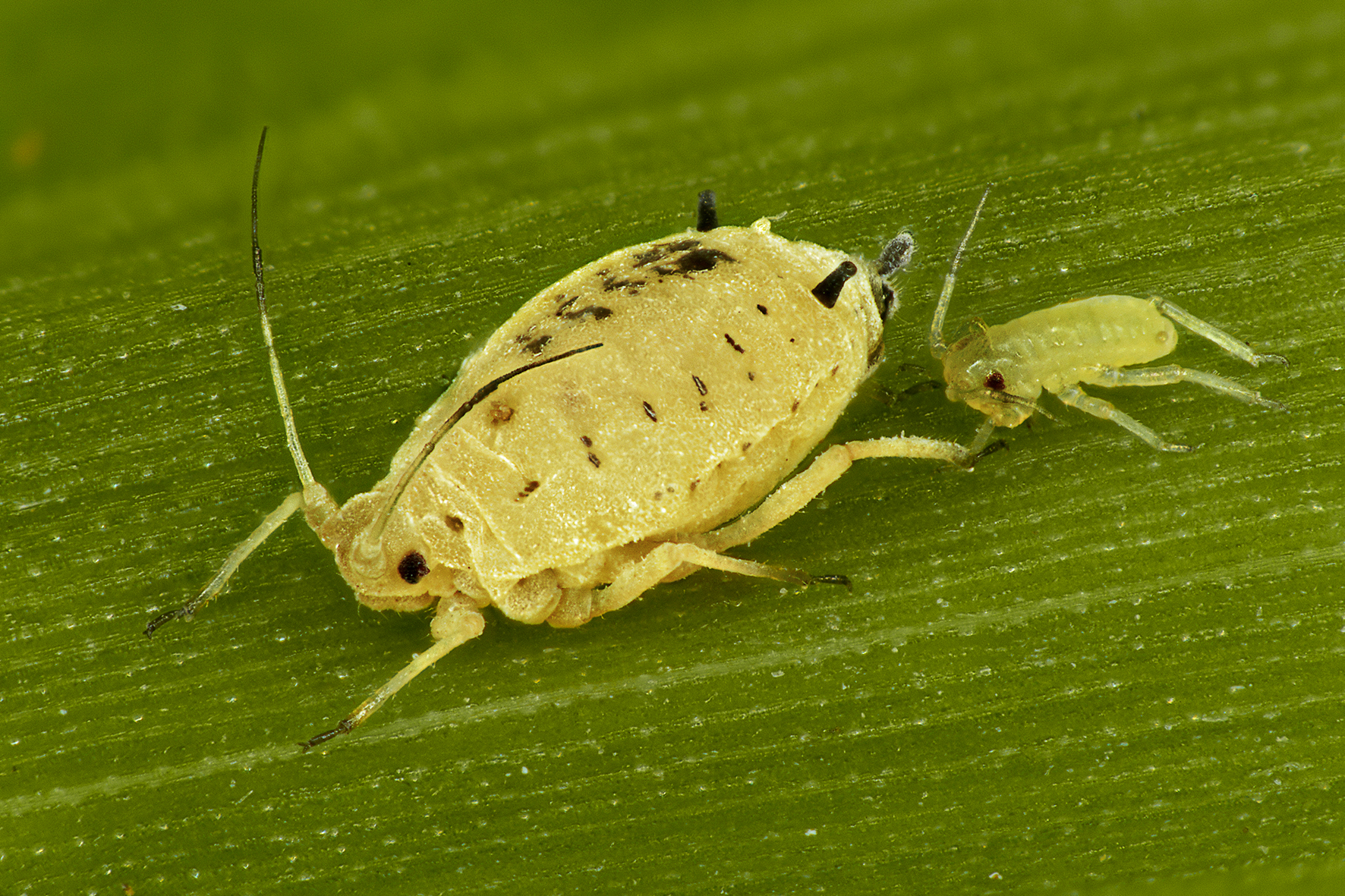

Supplement: S1 Fig — Apterous female left, neonate larva right (photography by A. Franck). (TIF) [file pone.0196124.s001.tif]

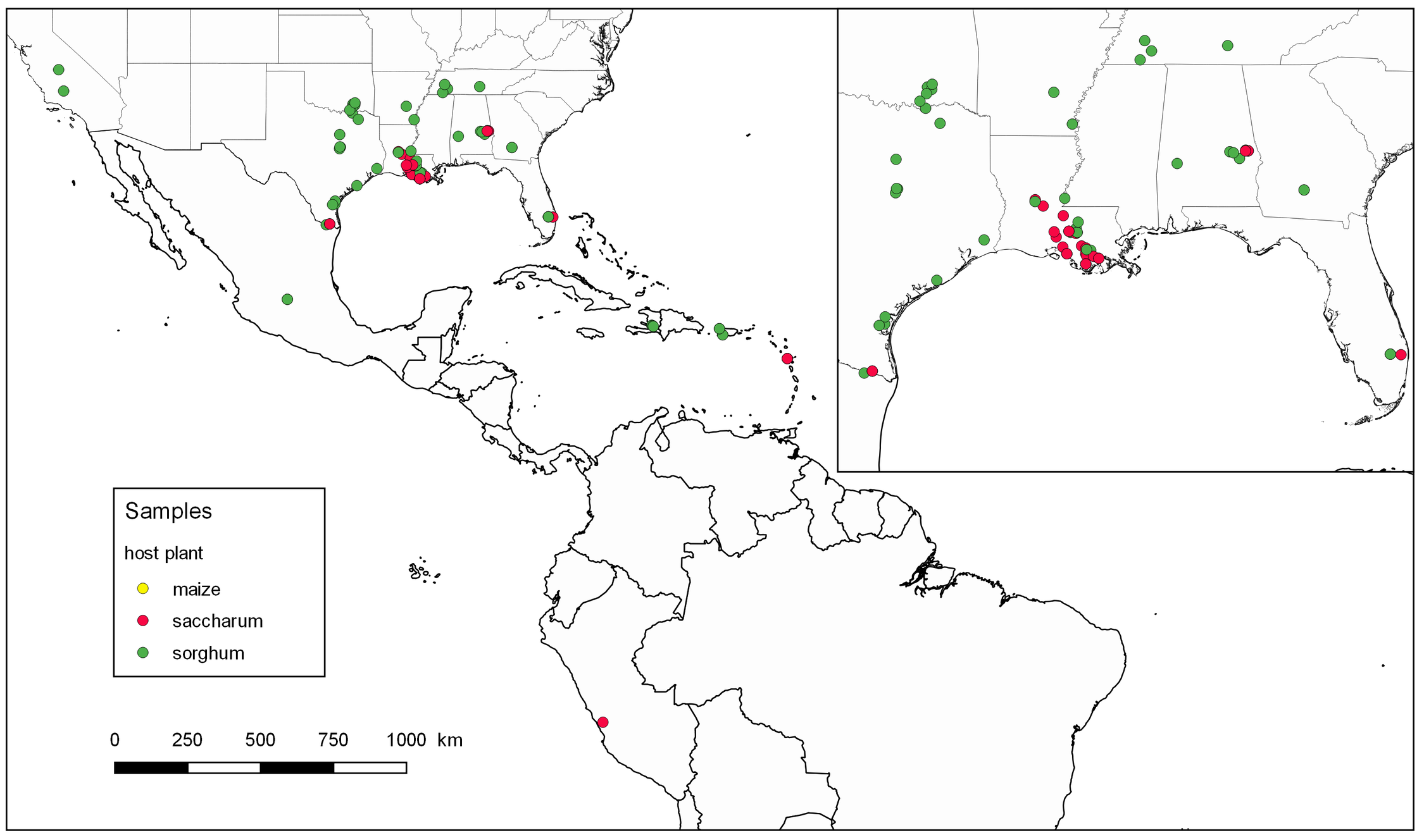

Supplement: S2 Fig — (TIF) [file pone.0196124.s002.tif]
